# Chestnut Brown Rot and *Gnomoniopsis smithogilvyi*: Characterization of the Causal Agent in Portugal

**DOI:** 10.3390/jof9040401

**Published:** 2023-03-24

**Authors:** Guilherme Possamai, Rosangela Dallemole-Giaretta, José Gomes-Laranjo, Ana Sampaio, Paula Rodrigues

**Affiliations:** 1 Centro de Investigação de Montanha (CIMO), Instituto Politécnico de Bragança, Campus de Santa Apolónia, 5300-253 Bragança, Portugal; 2 Laboratório Associado para a Sustentabilidade e Tecnologia em Regiões de Montanha (SusTEC), Instituto Politécnico de Bragança, Campus de Santa Apolónia, 5300-253 Bragança, Portugal; 3 Campus de Pato Branco, UTFPR-Universidade Tecnológica Federal do Paraná, Pato Branco 85503-390, PR, Brazil; 4 Centre for the Research and Technology of Agro-Environmental and Biological Sciences (CITAB), University of Trás-os-Montes e Alto Douro (UTAD), Quinta de Prados, 5000-801 Vila Real, Portugal; 5 Laboratório Associado Instituto para a Inovação, Capacitação e Sustentabilidade da Produção Agroalimentar (Inov4Agro), University of Trás-os-Montes e Alto Douro (UTAD), Quinta de Prados, 5000-801 Vila Real, Portugal

**Keywords:** *Castanea sativa*, *Gnomoniopsis castanea*, Koch postulates, phytopathogen

## Abstract

Sweet chestnut (*Castanea sativa* Miller) is a nutritious food with high social and economic impacts in Portugal. The fungus *Gnomoniopsis smithogilvyi* (syn. *Gnomoniopsis castaneae*) is the causal agent of chestnut brown rot, and is currently considered one of the major threats to the chestnut production chain worldwide. Considering the lack of knowledge on both the disease and the causal agent in Portugal, studies were conducted in an attempt to develop the necessary control strategies towards the mitigation of the disease in a timely way. Isolates of *G. smithogilvyi* were selected from three varieties of chestnut from the northeast of Portugal, and were characterized at the morphological, ecophysiological and molecular levels. Tests of pathogenicity and virulence were also developed. *Gnomoniopsis smithogilvyi* was confirmed as the causal agent of brown rot disease in Portuguese chestnut varieties, which showed high susceptibility. The fungus showed high adaptability to chestnut substrates. The Portuguese isolates of *G. smithogilvyi* are morphologically and genetically similar to those from other countries, even though some physiological variability was observed among them.

## 1. Introduction

The fruits of the European chestnut (*Castanea sativa* Miller) play an important role in human nutrition, especially for populations in the Mediterranean basin [1]. The chestnut also represents a relevant economic contribution to many European countries [2], such as Portugal, especially in the northeastern region of Trás-os-Montes, which is responsible for 88% of the Portuguese chestnut area and 82% of the production [3]. The region holds three chestnut protected denominations of origin (PDOs)—Terra Fria, Soutos da Lapa and Padrela. Among the 13 registered varieties in the region, three are of high productive and commercial significance—Longal, Judia and Martaínha [4,5]. Longal is highly distributed in the region and is considered the best variety for transformation, while Judia and Martaínha are preferably consumed fresh due to their higher caliber. Due to the nutritional characteristics of the sweet chestnut, combined with its high water content, the fruits are a substrate conducive to the development of fungi that cause post-harvest rot, affecting quality and, consequently, the commercial value of the chestnut.

Over the last decades, a sharp increase in the incidence of chestnuts presenting rot, with symptoms atypical to those of diseases that normally affect the fruits, has been observed by producers in Australia and New Zealand, and in some regions of Europe, such as Italy and France [6,7,8,9,10]. In 2012, the causal agent of this new chestnut fruit rot was attributed to the fungus *Gnomoniopsis smithogilvyi* L.A. Shuttlew, E.C.Y. Liew & D.I. Guest (2012) (syn. *Gnomoniopsis castaneae* G. Tamietti 2012), described as a new phytopathogenic fungal species.

*Gnomoniopsis smithogilvyi* infects the nut kernel, developing into browning and necrosis of the endosperm and embryo [11], and resulting in what is called brown chestnut rot. The disease is mainly expressed after harvest and it is cryptic, as healthy-looking nuts on the surface are rotten internally. The infection is initially asymptomatic, so it is assumed that the fungus colonizes chestnut tissues as an endophyte, becoming pathogenic as the nuts ripen. At early infection stages, the slightly moldy or parasitized nuts are not easily differentiated from the good ones, and infection is detected only when they are processed or consumed. Floral infection by ascospores or conidia is considered to be the main route of infection, but other infection paths, such as inoculum entrance via shell defects, are not ruled out [12]. The fungus shows the capacity to persist saprophytically in the burs and other residues on the ground, which represent the main reservoir for the formation of perithecia and subsequent release of infectious spores [9,11,13]. There is still a lack of knowledge on the potential of airborne inoculum and its dispersal pattern in time and space, as well as on the role of the climate in the infection patterns [14].

Currently, *G. smithogilvyi* is considered the main emerging fungus causing rot in chestnuts over a vast geographic area covering three continents. In Italy, Visentin et al. [9] reported an increase in the frequency of the isolation of this fungus in chestnuts from 29.2 ± 22.7% in 2008 to 74.6 ± 9.4% in 2011. Maresi et al. [15] also reported a 49% incidence of brown rot caused by this pathogen. In Australia and Switzerland, in the years 2013 and 2015, an incidence of chestnut rot of 72 and 91% was reported, respectively [10,16]. In Portugal, farmers started reporting an “unknown” rot in 2018, for which special evidence was gathered in 2019, and it is currently considered to be responsible for up to 80 or 90% of chestnut production losses in some regions of the country (non-published data). This fungus has now been isolated, identified, characterized and reported in chestnuts from the United Kingdom [17], United States of America [18], Chile [19], Spain [20], Ireland [21] and Turkey [22]. In Portugal, the first reports of the isolation of *G. smithogilvyi* from pre-harvest chestnuts were in 2017 [23] and in 2021 [24], and in post-harvest chestnuts in 2019 [25]. Although no extensive surveys about the incidence of *G. smithogilvyi* have been carried out in Portugal, Rodrigues et al. [25] reported a 6.4% incidence in stored chestnuts that had already been visually selected, with rejected chestnuts showing as much as 40% infection.

A small number studies reported the presence of *G. smithogilvyi* in Portugal and established its relation to the chestnut brown disease, but did not characterize the fungus in terms of virulence, ecophysiology and genetic relatedness to other isolates from different geographic origins. Due to the lack of knowledge and studies on Portuguese isolates from this phytopathogenic agent and its behavior in Portuguese chestnut varieties, the objectives of this work were to comprehensively morphologically, ecophysiologically and molecularly characterize three selected isolates of *G. smithogilvy* from three Portuguese varieties of *C. sativa*, Judia, Longal and Martaínha, to determine their pathogenicity and virulence in chestnuts of these varieties and to evaluate the natural infection of the fruits of these varieties by this fungus.

## 2. Materials and Methods

### 2.1. Biological Material

The assays were carried out in the production year of 2019/2020, using three varieties of sweet chestnut (*C. sativa*)—Judia, Longal and Martaínha. These varieties were selected based on their economic importance and generalized geographic distribution in the region of Trás-os-Montes [4,5] and their apparent susceptibility to fungal growth as reported by Rodrigues et al. [25]. The chestnut samples were collected from a local industry in paper bags, transported to the Mycology Laboratory of CIMO (Centro de Investigação de Montanha), Instituto Politécnico de Bragança, Portugal, and preserved at 4 °C until analysis (max. 48 h). 

The isolates of *G. smithogilvyi* used in the present study were previously collected from local chestnuts, cultivar Longal (isolates G1 and G2) and Judia (isolate G3), from the production year 2018/2019, and identified molecularly as described in Rodrigues et al. [25]. Isolates G1, G2 and G3 were deposited in the culture collection “Micoteca of Universidade do Minho (MUM)”, Braga, Portugal, with the codes MUM 20.139, MUM 20.140 and MUM 20.141, respectively. All isolates were grown on potato dextrose agar (PDA, BioLife, Milan, Italy) for 5 days at 25 °C before analysis.

### 2.2. Tests of Pathogenicity and Virulence of G. smithogilvyi Isolates

#### 2.2.1. Preparation of Chestnuts

Chestnuts from the three varieties were carefully inspected for defects, such as discoloration, broken pericarp (shell), irregular tenderness, signs of pest infestation and fungal infection. Only apparently healthy nuts were selected. Nuts were washed with running tap water, surface-disinfected with 10% commercial bleach (0.5% sodium hypochloride) for 10 min, rinsed twice with sterilized water and blot-dried in sterile paper towels in a laminar flow hood. After that, the nuts were submitted to UV light for 20 min.

#### 2.2.2. Preparation of Spore Suspensions

For this assay, the isolates G1 and G2 were used from 7-day-old PDA cultures grown at 25 °C. Conidiomata were collected from the plate using a sterile loop and homogenized by vortexing in sterile water with 0.1% Tween 80. Spore concentrations were determined using a Neubauer counting chamber, and were adjusted to 2 × 10^7^ spores/mL in sterile water with 0.1% Tween 80.

#### 2.2.3. Inoculation and Incubation

For each binomium fungal isolate and chestnut variety, three different methods of inoculation were tested. In all methods, chestnuts were placed in 250 mL glass jars (three chestnuts each), and 20 µL of the spore suspension was used as inoculum. The inoculation methods were as follows: (i) intact chestnuts were superficially inoculated (on the pericarp) in three spots—near the hilum, near the embryo and in the center; (ii) chestnuts were perforated with a sterile needle in three spots, and inoculated on the whole; (iii) chestnuts were longitudinally cut in half with a sterile scalpel, and the two halves of the endosperm were inoculated. For each treatment and each variety, six non-inoculated chestnuts were used as the control. In total, 48 jars and 144 chestnuts were used. Chestnuts were incubated in the dark at 25 °C for 15 days.

#### 2.2.4. Evaluation of Infection Rate in Chestnuts Inoculated with *G. smithogilvyi*

Chestnuts were longitudinally cut and the fungal infection rate was registered in terms of incidence, and the severity of infection rate was registered in terms of percentage of chestnut area with visible rot as proposed by Donis-González et al. [26]: Level 0 (L0): no visible rot; Level 1 (L1): 1–25% of the chestnut area with rot; Level 2 (L2): 26–50%, Level 3 (L3): 51–75% and Level 4 (L4): 76–100%. Incidence was registered as the percentage of infected chestnuts. The severity was then calculated from these infection levels using the McKinney index [27]. This index takes into account the intensity of the rot (severity), its frequency and the maximum possible value, according to the equation

(1) MI=[∑((d×f))/(N×D)]×100

where d is the category of rot intensity scored for chestnuts, f is the rot frequency, N is the total number of examined chestnuts (healthy and rotted) and D is the highest category of rot intensity that occurred.

#### 2.2.5. Verification of Koch’s Postulates

The Koch’s postulates were verified in the inoculated chestnuts that showed the development of brown rot. The rot-causing agent was re-isolated from each infected fruit in PDA Petri dishes and was incubated for 7 days at 25 °C in the dark, after which it was morphologically identified and compared with the original cultures.

### 2.3. Natural Incidence of G. smithogilvyi in Chestnuts

This assay was performed to evaluate the endophytic incidence of the fungus. For this, 36 glass flasks and 108 chestnuts (36 of each variety) were used. Chestnuts were selected and surface-disinfected as previously described, and were divided into four sets (for each variety). They were longitudinally cut and incubated for 7 days, and the intact chestnuts were incubated for 7, 14 and 21 days. All treatment groups were incubated at 25 °C, and carried out in triplicate (total of 9 chestnuts per treatment for each chestnut variety). After the incubation period, the intact chestnuts were longitudinally cut and the fungal infection rate was registered in terms of percentage of chestnut area with visible rot [26], as previously described.

### 2.4. Morphological and Cultural Characterization of the Isolates

#### 2.4.1. Biological Material and Culture Media

In this assay, the isolates G1, G2 and G3 were used. Growth parameters were evaluated in three media: potato dextrose agar (PDA, Biolife, Italy), malt extract agar (MEA, Himedia, Mumbai, India) and chestnut medium (MC). PDA and MEA were used as standard media for comparison purposes with previously reported data [9,11]. The media were prepared following the manufacturers’ instructions. MC was used as a model medium to mimic the chemical and nutritional conditions of chestnuts to better understand the isolates’ ecophysiology, in particular, the adaptability to chestnut as a substrate. For the preparation of MC, fresh and healthy chestnuts (balanced mix of the three varieties Judia, Longal and Martaínha) were cooked for 15 min in a microwave, shelled and blended using a kitchen blender in the proportion of 200 g per 1 L of distilled water. Agar was added at 2%, and the medium was autoclaved for 121 °C for 15 min.

#### 2.4.2. Inoculation and Growth Parameters

Spore suspensions of each isolate were prepared at 3 × 10^7^ spores/mL as previously described. Petri dishes with a 9 cm diameter containing 20 mL of each medium were central point-inoculated with 20 µL of each spore suspension (in triplicate), and were incubated at 5, 12, 20, 25, 30 and 35 °C for 9 days in the dark. 

Fungal growth was measured in two directions daily for nine days or until the colony achieved the maximum diameter (8.5 cm). From these data, the following parameters were calculated: (i) lag phase (λ, in days), corresponding to the number of days from inoculation until mycelial growth was visible and (ii) mean growth rate (κ, day^−^^1^), calculated from the slope of the regression line in the exponential phase from the colony diameter plotted against incubation time. Spores were counted for each culture medium 9 days after inoculation, but only for the 25 °C incubation. For this, spores were collected from the entire plate by flooding the medium with 10 mL of 0.9% NaCl with 0.1% Tween 80, and were counted with a Neubauer hematocytometer.

#### 2.4.3. Measurement of Conidia and Conidiomata

At least 100 conidia and 50 conidiomata of 7-day-old cultures of each culture medium were measured transversally and longitudinally using a microscope and a stereomicroscope, respectively, both coupled to Leica Application Suite V4 (LAS V4.12) software. Measures are expressed as (minimum)–mean–(maximum) (length × width).

### 2.5. Molecular Characterization of the Isolates

Genomic DNA of the isolates was extracted following the SDS protocol described by Rodrigues et al. [28]. A portion of the internal transcribed spacer (ITS) of the ribosomal RNA locus (600 bp amplicons) and of the gene encoding the translation elongation factor 1α (TEF-1α; 1000 bp amplicons) were amplified through PCR using the primer pairs ITS1 (5′-CTTGGTCATTTAGAGGAAGTAA-3′)/ITS4 (5′-TCCTCCGCTTATTGATATGC-3′) [29], and EF1-728F (5′-CATCGAGAAGTTCGAGAAGG-3′)/EF1-1567R (5′-ACHGTRCCRATACCACCRATCTT-3′) [30,31]. The reactions occurred in a final volume of 25 μL using the following protocol: 94 °C, 3 min; 94 °C, 30 s; 55 °C, 30 s; 72 °C, 2 min (35 cycles); 72 °C, 10 min. The PCR products were purified using the GF-1 purification kit (Vivantis, Selangor Darul Ehsan, Malaysia) following the manufacturer’s instructions, and were then sequenced in both directions by STAB VIDA (Caparica, Portugal), using ABI 3730xl equipment (Applied Biosystems, Waltham, MA, USA). The sequences were processed manually, and a consensus sequence was created using the BioEdit v7.0.9 and Sequencher^®^ Version 4.9 (Demo version) programs. The consensus sequences were compared with the ones in the GenBank NCBI (National Centre for Biotechnology Information) database (http://www.ncbi.nlm.nih.gov/, accessed on 15 October 2020) using the BLASTn (nucleotide Basic Local Alignment Search Tool) algorithm. The sequences were deposited in GenBank with the accession numbers MW165483—MW165485 (ITS) and MW170363—MW170365 (TEF1-α gene).

Alignments were generated using ClustalX [32]. Phylogenetic trees were constructed using MEGA11 [33], using the neighbor-joining method [34] and the Jukes–Cantor parameters method [35] with 1000 bootstrap replicates. The evolutionary distances were computed using the maximum composite likelihood method [36]. Sequences of *G. smithogilvyi* isolates from different geographic regions obtained in previous studies were retrieved from GenBank and used for comparison.

### 2.6. Statistical Analysis

Since both the severity and incidence raw and transformed data failed the normality criteria, a non-parametric analysis was applied using the Kruskal–Wallis test followed by the post hoc Mann–Whitney test. The level of significance was established for *p* < 0.05. Tests were run with the IBM SPSS^®^ Statistics v. 22 software.

In the ecophysiology assays, the logarithm (Ln) of the diametral growth was used to determine the mean growth rate (κ) and the R^2^ value was used to determine the experimental data fitting the model. All the growth rate values were compared to determine significant differences between conditions using GraphPad v. 8.0. Since the data failed the normality premise, a non-parametric analysis was applied using the Kruskal–Wallis test followed by multiple comparisons to evaluate the influence of culture media, isolates and temperature on the growth parameters lag phase, mean growth rate and sporulation.

## 3. Results

### 3.1. Natural Incidence of G. smithogilvyi in Chestnuts

The results for the natural incidence and severity (as given by the McKinney index) of *G. smithogilvyi* in chestnuts are presented in Table 1. Given the high variability of incidence, no significant differences were observed between varieties (*p* = 0.721) or incubation conditions (*p* = 0.920).

### 3.2. Tests of Pathogenicity and Virulence of G. smithogilvyi Isolates

**Figure 1:**
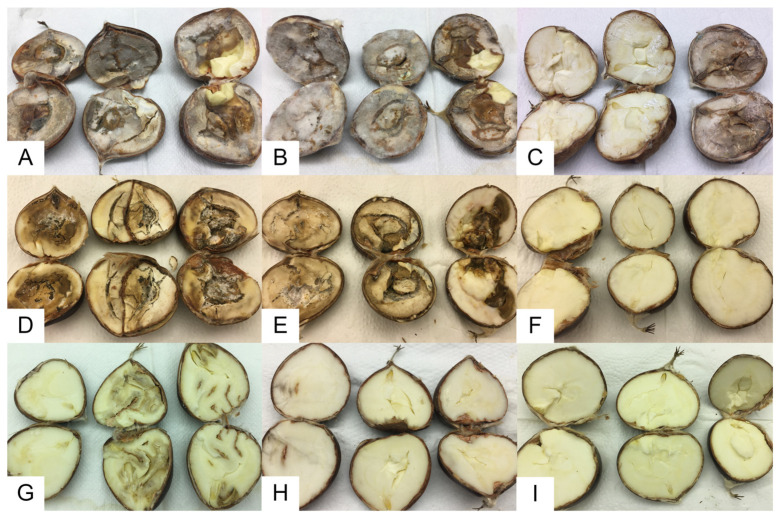
Chestnuts inoculated with *G. smithogilvyi* and observed after 15 days of incubation at 25 °C. Cut chestnuts inoculated with strains G1 (**A**) and G2 (**B**), and not inoculated (**C**). Perforated chestnuts inoculated with strains G1 (**D**) and G2 (**E**), and not inoculated (**F**). Intact chestnuts inoculated with strains G1 (**G**) and G2 (**H**), and not inoculated (**I**).

Overall, there was no significant difference in either incidence or severity between chestnut varieties (*p* = 0.596) and fungal strains (*p* = 0.774) in inoculated chestnuts (Table 2; Figure 1). Differences were found between treatments for both parameters (*p* < 0.0001). After the Mann–Whitney tests, no significant difference was detected in rot incidence and severity between cut and perforated fruits (*p* = 1.000), where the fungus had direct contact with the endosperm. In contrast, in intact fruits, the fungus caused significantly lower disease incidence and severity, resulting in significant differences between the treatments intact/cut and intact/perforated (*p* = 0.000). In the exposed cotyledons (cut chestnuts), the fruits were fully infected (100% of infected area) by the fungus, which was always re-isolated, following the Koch’s postulates.

### 3.3. Morphology and Ecophysiology of G. smithogilvyi

**Figure 2:**
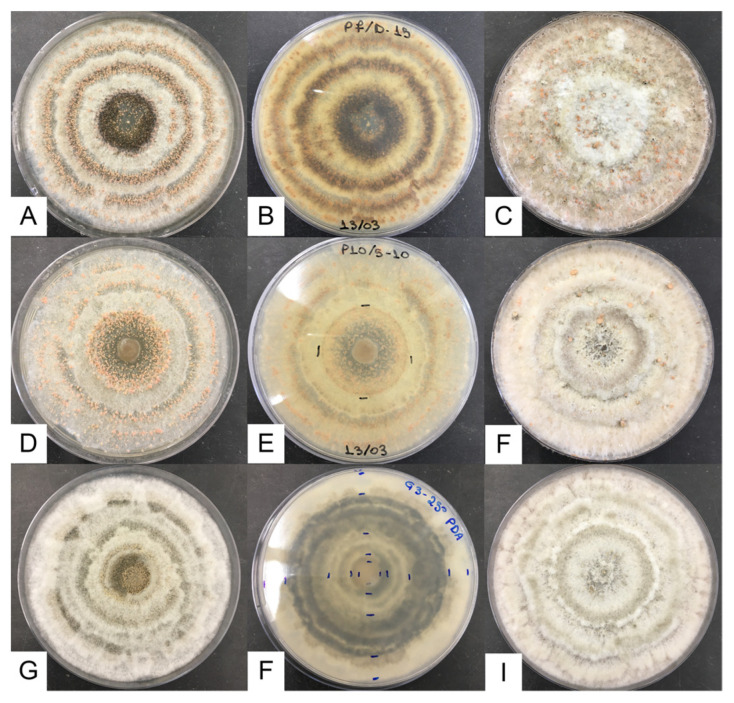
*G. smithogilvyi* colonies after 7 days of incubation at 25 °C. Top: isolate G1: (**A**) obverse in PDA, (**B**) reverse in PDA, (**C**) obverse in CM. Middle: isolate G2: (**D**) obverse in PDA, (**E**) reverse in PDA, (**F**) obverse in CM; Bottom: isolate G3: (**G**) obverse in PDA, (**H**) reverse in PDA, (**I**) obverse in CM.

**Figure 3:**
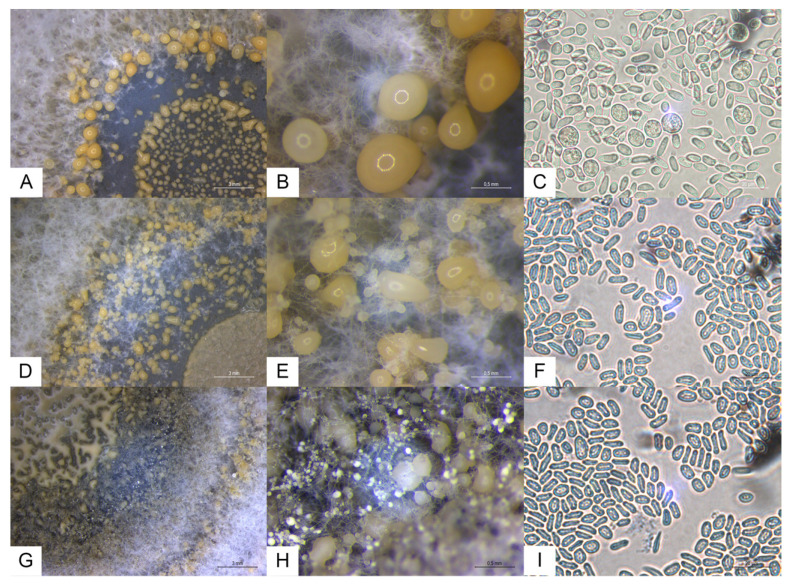
Morphological details of *G. smithogilvyi* isolates G1 (top line), G2 (middle line) and G3 (bottom line) in PDA, after 7 days of incubation at 25 °C. (**A**,**D**,**G**) Colony center, rich in conidiomata distributed in concentric rings. (**B**,**E**,**H**) Details of conidiomata. (**C**,**F**,**I**) Conidia.

Under standard conditions, *G. smithogilvyi* produced a relatively dense and woolly mycelium, with a beige color, diffuse to regular margins, with concentric rings of yellowish conidiomata on the obverse and dark on the reverse of the colony (Figure 2). In CM, the morphology of the fungus differed from that in PDA by being denser and more aerial, with fewer but bigger conidiomata showing an irregular distribution (no evident ring distribution). Conidiomata were abundant, globose to subglobose, viscous, with colors varying from cream to light orange, mostly yellow to light orange (Figure 3).

The conidia from *G. smithogilvyi* showed variable shapes and sizes, mostly straight or slightly curved, cylindrical or ellipsoidal (Figure 3C,F,I). In general, the average conidia size for the three isolates did not show major differences between culture media. In contrast, the size of the conidiomata showed significant differences between both culture media and isolates (*p* < 0.05), despite the high size diversity observed (Table 3). On the other hand, the substrate significantly influenced the size of the conidiomata, which were significantly bigger in CM (average of the three strains 435 µm) than in the other tested media (209 µm in PDA; and 67 µm in MEA).

**Figure 4:**
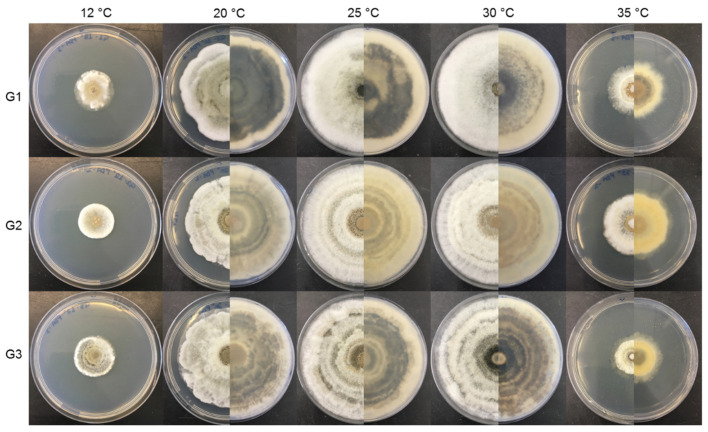
Growth of the three isolates of *G. smithogilvyi* (G1, G2 and G3) at five different temperatures (12, 20, 25, 30 and 35 °C) in PDA, after 7 days of incubation (obverse/reverse).

**Figure 5:**
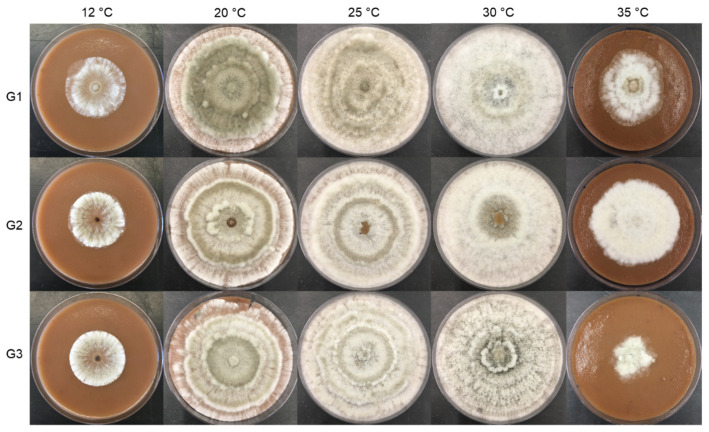
Growth of the three isolates of *G. smithogilvyi* (G1, G2 and G3) at five different temperatures (12, 20, 25, 30 and 35 °C) in CM, after 7 days of incubation.

The three isolates of *G. smithogilvyi* presented intraspecific differences in colony morphology and growth (Figure 4 and Figure 5) when grown in the media PDA and CM at different temperatures (12, 20, 25, 30 and 35 °C).

**Figure 6:**
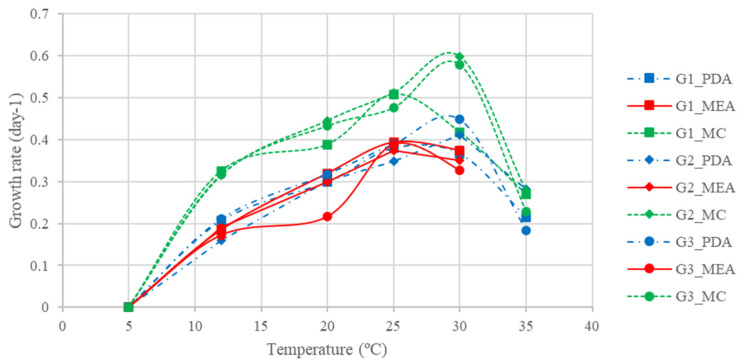
Growth rate of *G. smithogilvyi* (isolates G1, G2 and G3) in potato dextrose agar (PDA), and chestnut medium (CM), at temperatures from 5 to 35 °C, and malt extract agar (MEA) at temperatures from 5 to 30 °C.

Figure 6 shows the growth rates of the three isolates in each of the three culture media for the temperature range 5 to 35 °C (MEA was not tested at 35 °C). Even though no significant differences were observed for each isolate between the temperatures 20, 25 and 30 °C (*p* > 0.05), optimal growth occurred between 25 and 30 °C with growth rates of 3.5 to 6 mm/day. The growth rate was similar between PDA and MEA (*p* = 1.000), but significantly higher in CM (*p* < 0.05) for all isolates.

**Figure 7:**
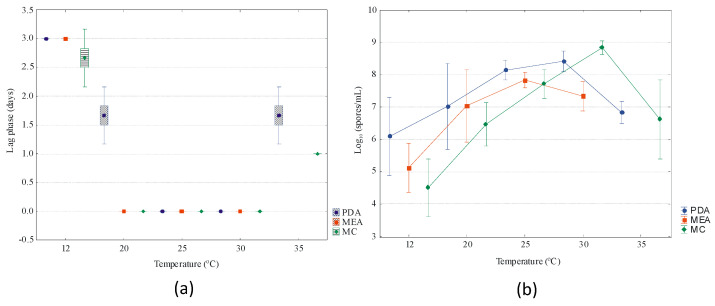
(**a**) Lag phase (in days) and (**b**) spore counts for *G. smithogilvyi* in potato dextrose agar (PDA), and chestnut medium (CM), at temperatures from 12 to 35 °C, and in malt extract agar (MEA) at temperatures from 12 to 30 °C.

The results for lag phase and conidia production are presented in Figure 7. The lag phase was not significantly different between the isolates for the same temperature, considering each culture medium (*p* > 0.05). Within the 20 to 30 °C range, the fungus started to grow immediately after inoculation (lag phase = 0 days), while at 5, 12 and 35 °C, the lag phase was significantly longer (*p* = 0.000). It is noteworthy that for these suboptimal temperatures, the lag phase was significantly lower in CM when compared with PDA and MEA.

In terms of conidia production, there was no significant difference between the culture media (*p* = 0.379), but temperature significantly influenced this parameter, ranging from 3.5 log10 spores/mL (MC, 12 °C) to 9 log10 spores/mL (MC, 30 °C). It is noted that the isolates produced the lowest amount of spores in CM for all temperatures except at 30 °C.

### 3.4. Molecular Characterization

**Figure 8:**
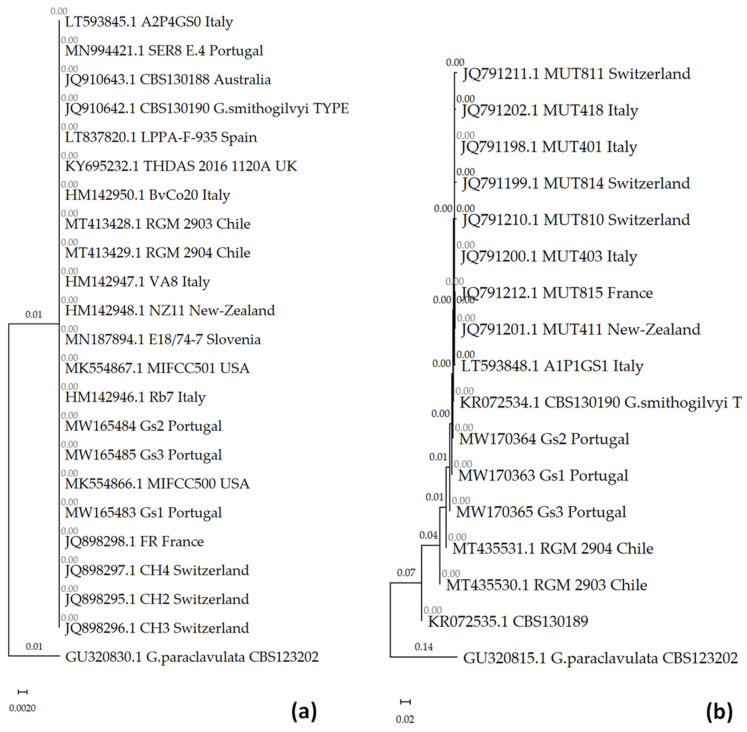
Phylogenetic tree for *G. smithogilvyi* isolates. (**a**) ITS region for 23 sequences, in a total of 513 nucleotide positions; (**b**) TEF1-α partial gene for 17 sequences, in a total of 1005 nucleotide positions. The optimal tree is shown, as inferred using the neighbor-joining method [34]. The tree is drawn to scale, with branch lengths (above the branches) in the same units as those of the evolutionary distances used to infer the phylogenetic tree. The evolutionary distances, computed using the maximum composite likelihood method [36], are in the units of the number of base substitutions per site. All ambiguous positions were removed for each sequence pair (pairwise deletion option).

The molecular analysis of the *G. smithogilvyi* isolates was performed on the basis of the ITS region and the TEF1-α gene (Figure 8). In this analysis, sequences of *G. smithogilvyi* isolates with different geographical origins were retrieved from GenBank (19 ITS and 13 TEF1-α sequences). *Gnomoniopsis paraclavulata* Sogonov was used as the tree root for both molecular markers. The phylogenetic analysis of the *G. smithogilvyi* isolates showed a very close relation between the isolates from this study and those retrieved from GenBank, including the type of strain. This is in agreement with other studies [15,16], where no differences were detected between isolates with worldwide geographic distribution.

## 4. Discussion

In recent years, many studies have been conducted on the fungal pathogen *G. smithogilvyi*, mostly from countries where the chestnut is a significant commercial product [37,38,39,40]. In Portugal, Coelho and Gouveia [23] described *G. smithogilvyi* as the causal agent of brown chestnut rot for the first time, and it has been isolated from Portuguese chestnuts at frequencies ranging from 9.3% to 15.3% [23,25,41], but no studies have been previously reported on the morphology and ecophysiology of the isolates from Portugal and their comparison to the ones isolated from other geographical areas.

In the present study, *G. smithogilvyi* was isolated from asymptomatic chestnuts from three *C. sativa* Portuguese varieties—Judia, Longal and Martaínha confirming its endophytic behavior, as also reported by others from various geographic origins [9,12,15,16]. No significant differences were observed for incidence between the three varieties (Table 1). The Koch postulates confirmed *G. smithogilvyi* to be the causal agent of chestnut brown rot. Pathogenicity and virulence assays revealed that all of the tested *G. smithogilvyi* isolates were capable of colonizing the chestnut tissue, showing high virulence and causing intense symptoms of brown rot in all chestnut varieties (Figure 1). These assays also revealed the role of the outer shell in controlling the development of the disease, mostly in the case where infection occurs from external propagules. Even though the fungus shows endophytic behavior, infections may also occur after harvest if the shell is ruptured. These assays demonstrate the importance of chestnut management practices at both pre- and post-harvest stages of chestnut production, as suggested by Silva-Campos et al. [39].

Our study provided detailed data on the main morphological and ecophysiological characteristics of three isolates of *G. smithogilvyi*. The colony showed a yellowish tone and the presence of numerous yellow to orange conidiomata (Figure 2 and Figure 3), typical of the genus *Gnomoniopsis* [42]. The Portuguese isolates were similar to those from Australia, Italy, New Zealand and India [9,11,13], in terms of colony aspect as well as conidia and conidiomata size and morphology when grown in PDA. In contrast, in MEA, the conidiomata of the isolates were significantly smaller than those from Australia [11]. Additionally, the average growth rate at 25 °C in MEA was higher (approximately 4 mm/day) than that reported by Visentin et al. [9] (0.8 to 1.0 mm/day). This difference may be related to the method of calculation, since in our study, the growth rate considered only the logarithmic phase. 

From a molecular perspective, our isolates showed a very high degree of genetic similarity (>99%) for the ITS region to the isolates from Australia, Chile, Slovenia, Spain, the USA, France, Italy, New Zealand, the United Kingdom and Switzerland [9,17,18,19,43,44]; (Figure 8). The TEF1-α sequence showed a higher diversity among isolates, with the Portuguese ones closer to those from Chile and Australia. Silva-Campos et al. [40] developed and validated a multiplex PCR assay for *G. smithogilvyi* detection, and observed a very low level of genetic divergence between Australian isolates and those from other geographic origins.

In a study by Lione et al. [14], the authors reported that the production of conidia and other growth parameters by *G. smithogilvyi* are dependent on various factors such as the biological features of the isolate and the climatic and environmental conditions. In fact, in the present study, the ecophysiological studies were conducted in three different culture media, PDA, MEA and CM. While PDA and MEA were the media used for fungal characterization by others [9,11,42], a chestnut-based medium mimicking the substrate of interest had not been tested previously. By comparing growth parameters in CM with those in PDA and MEA, it was clear that *G. smithogilvyi* shows an optimal adaptation to chestnut, namely lower lag phase, higher growth rate and higher mycelial growth (lower conidia production). It is also interesting to note that growing in MC at 30 °C stimulates the production of conidia, which might reflect an adaptation to stressful temperatures. This effect may have a negative impact on the virulence of the fungus, which could potentially increase under the conditions of climate change already in place.

## Figures and Tables

**Table 1:** Natural incidence (%) and severity (McKinney index) of brown rot caused *by G. smithogilvyi* in chestnut varieties Longal, Judia and Martaínha (n = 9 ± SD).

	Incidence (%)			Severity		
Treatment	Longal	Judia	Martaínha	Longal	Judia	Martaínha
Cut (7 days)	56 ± 38	44 ± 38	33 ± 33	0.14	0.08	0.08
Intact (7 days)	22 ± 38	11 ± 19	11 ± 19	0.06	0.03	0.03
Intact (14 days)	11 ± 19	11 ± 19	22 ± 19	0.03	0.03	0.08
Intact (21 days)	11 ± 19	22 ± 19	0	0.03	0.14	0.00

**Table 2:** Incidence (%) and severity (McKinney index) of brown rot caused by *G. smithogilvyi* in chestnut varieties Longal, Judia and Martaínha artificially infected with two strains of the fungus, G1 and G2 (n = 6). Different letters mean significant differences between methods of inoculation), after Kruskal–Wallis non-parametric analysis followed by the post hoc Mann–Whitney test.

		Incidence (%)			Severity		
Isolate	Type of Inoculation	Longal	Judia	Martaínha	Longal	Judia	Martaínha
Not inoculated	Cut	0 ^a^	0 ^a^	17 ^a^	0.00 ^a^	0.00 ^a^	0.17 ^b^
	Perforated	50 ^b^	17 ^a^	50 ^b^	0.29 ^c^	0.08 ^a^	0.25 ^b^
	Intact	33 ^b^	17 ^a^	0 ^a^	0.13 ^b^	0.04 ^a^	0.00 ^a^
G1	Cut	100 ^a^	100 ^a^	100 ^a^	0.83 ^a^	1.00 ^a^	0.83 ^a^
	Perforated	100 ^a^	100 ^a^	100 ^a^	1.00 ^a^	0.92 ^a^	0.67 ^a^
	Intact	50 ^b^	17 ^b^	0 ^b^	0.20 ^b^	0.04 ^b^	0.00 ^b^
G2	Cut	100 ^a^	100 ^a^	100 ^a^	0.92 ^a^	0.92 ^a^	1.00 ^a^
	Perforated	100 ^a^	100 ^a^	100 ^a^	0.79 ^a^	0.58 ^a^	0.83 ^a^
	Intact	17 ^b^	17 ^b^	0 ^b^	0.17 ^b^	0.17 ^b^	0.00 ^b^

**Table 3:** Conidiomata size (n = 50) and conidia length and width (n = 100) of *G. smithogilvyi* isolates G1, G2 and G3 in potato dextrose agar (PDA), malt extract agar (MEA) and chestnut medium (CM). Min–Mean–Max (SD: standard deviation).

Parameter	G1	G2	G3
Conidiomata (µm)			
PDA	74-220-474 (89.0) ^aA^	172-301-393 (54) ^bA^	22-107-350 (99) ^cA^
MEA	24-79-175 (39.4) ^aB^	26-70-119 (21) ^aB^	24-52-109 (20) ^bB^
CM	278-501-757 (114) ^aC^	174-391-643 (122) ^bA^	206-413-633 (103) ^bC^
Conidia Length (µm)			
PDA	4.8-6.3-8.1 (0.8) ^aA^	5.4-6.9-8.3 (0.7) ^bA^	4.9-6.7-8.1 (0.7) ^bA^
MEA	4.4-5.7-7.2 (0.7) ^aB^	5.2-7.0-8.8 (0.8) ^bA^	5.3-6.7-8.1 (0.7) ^cA^
CM	4.9-6.4-7.6 (0.6) ^aA^	5.8-6.9-8.3 (0.6) ^bA^	4.9-6.7-8.7 (0.9) ^bA^
Conidia Width (µm)			
PDA	2.0-2.6-3.2 (0.3) ^cAB^	1.9-2.8-3.4 (0.3) ^bA^	2.3-3.0-4.0 (0.4) ^aA^
MEA	2.2-2.7-3.1 (0.2) ^aA^	2.2-3.1-3.8 (0.4) ^bB^	2.1-2.9-3.6 (0.4) ^cA^
CM	1.8-2.5-3.2 (0.3) ^aB^	2.3-3.1-4.1 (0.4) ^bB^	1.7-2.3-3.0 (0.3) ^cB^

Lower case letters: comparison between isolates in the same culture medium. Upper case letters: comparison between media for the same isolate.

## Data Availability

Not applicable.
